# J-shaped relationship between cardiovascular risk and efficacy of intensive blood pressure reduction: A post-hoc analysis of the SPRINT trial

**DOI:** 10.1371/journal.pone.0240102

**Published:** 2020-10-01

**Authors:** Armin Attar, Fatemeh Nouri, Roham Borazjani, Mehrab Sayadi

**Affiliations:** 1 Cardiovascular Research Center, TAHA Clinical Trial Group, Shiraz University of Medical Sciences, Shiraz, Iran; 2 Students’ Research Committee, Shiraz University of Medical Sciences, Shiraz, Iran; International University of Health and Welfare, School of Medicine, JAPAN

## Abstract

**Background:**

In the 2017 ACC/AHA hypertension guidelines, a 10-year risk of more than 10% is considered for initiation of intensive blood pressure reduction. The current study aimed to determine which cut off limit of cardiovascular risk for starting intensive blood pressure reduction is beneficial.

**Design:**

A Secondary Analysis of Systolic Blood Pressure Intervention Trial (SPRINT).

**Methods:**

Data from the SPRINT Trial was obtained from the NHLBI Data Repository Center. In the SPRINT, non-diabetic participants with SBP of ≥ 130 mmHg were randomly assigned to intensive and standard treatment arms with SBP targets of < 120 and < 140 mmHg, respectively. This study analyzed data from non-diabetic participants less than 75 years of age without cardiovascular or chronic kidney disease. The primary composite outcome was myocardial infarction, and other acute coronary syndromes, stroke, heart failure, or death from cardiovascular causes. Cox regression models were used to examine the risk of the occurrence of the SPRINT primary composite outcome. To identify the relationship between BP values and the log hazards, natural cubic spline functions were performed.

**Results:**

In the analysis, 4292 patients were enrolled. The results demonstrated a clear J-shaped relationship between the effect of intensive blood pressure control and the risk of CVD events and 10-year Framingham cardiovascular risk levels at a cut-off limit of approximately <7%.

**Conclusions:**

This post-hoc secondary analyses of the SPRINT trial showed that a cut off value of more than 7% may be useful in selecting patients suitable for initiation of blood pressure reduction.

## Introduction

Hypertension is well known as a leading cause of cardiovascular diseases (CVDs) [1]. Many hypertension treatment guidelines have made a priority of treating blood pressure alone rather than treating blood pressure in association with cardiovascular disease risk [2, 3]. Cholesterol treatment guidelines, however, have replaced single-risk-factor treatments with absolute risk assessment to provide instruction for preventive therapies [4, 5]. The 2017 American College of Cardiology/American Heart Association (ACC/AHA) hypertension guidelines suggested that intensive blood pressure lowering be initiated in individuals with a 10-year risk of higher than 10%, a newly detected SBP of between 130 to 139 mmHg at baseline, and without a known CVD [6]. This recommendation is a cause for concern as it is not based on a clinical trial [7]. It is presumed that this suggestion is according to the results of the Systolic Blood Pressure Intervention Trial (SPRINT) trial [7]; However, almost all participants of the SPRINT trial were already hypertensive and on anti-hypertensive drugs [8].

The SPRINT trial revealed that intensive reduction of systolic blood pressure (SBP) can decrease the rate of cardiovascular events in non-diabetic participants with a high cardiovascular disease risk [8]. Specifically, the SPRINT did not assess the outcomes of intensive blood pressure control associated with total cardiovascular risks. Some studies have also shown that intensive blood pressure reduction in high risk patients has significant benefits [9–11]. Recently, a secondary analysis of the SPRINT indicated that intensive SBP reduction to a level below 120 mm Hg was beneficial for primary prevention of cardiovascular morbidity and mortality in non-diabetic patients with a cardiac risk above 10% [9]. In this study, a secondary analysis of 10-year Framingham cardiovascular risk levels and the risk of developing the primary composite cardiovascular outcome from the SPRINT trial was performed to determine which cut-off limit of cardiovascular risk for initiating intensive blood pressure control is helpful in preventing cardiovascular events.

## Methods

### Data collection

This study used data from the SPRINT trial, received from the National Heart, Lung, and Blood Institute (NHLBI) Biologic Specimen and Data Repository Information Coordinating Center.

### Study design and population

The study design of the SPRINT trial has been explained point by point elsewhere [8]. In brief, the randomized, controlled, open-label trial was conducted at 102 clinical sites on 9361 participants who had an SBP above 130 mmHg and increased cardiovascular disease risk. Patients were randomly assigned to one of the two treatment arms in this study: the control arm targeting an SBP <140 mmHg, or the intensive treatment arm with the target of SBP <120 mmHg. The inclusion criteria were an SBP between 130 mmHg and 180 mmHg, age ≥50 years, and elevated risk of cardiovascular events. Elevated cardiovascular risk was described as due to one or more of the following: 1) presence of CKD (excluding polycystic renal disease) with eGFR varying from 20 to lower than 60, computed by the Modification of Diet in Renal Disease (MDRD) formula; 2) according to the Framingham Cardiovascular Risk Score, a 10-year risk of more than 15% for CVD; 3) clinical or sub-clinical CVD excluding stroke; and 4) age at least 75 years. Participants with diabetes mellitus or a previous history of stroke were excluded. Due to the data clarification stated by NHLBI Biologic Specimen and Data Repository Information Coordinating Center, some participants were enrolled in the SPRINT who had an intermediate Framingham risk score or even a low risk score. In the current study, participants who had a baseline chronic kidney disease or cardiovascular disease and those who were aged 75 years or older were excluded. Using the 10-year Framingham cardiovascular risk scores, patients were divided into low risk (risk < 10%), intermediate risk (risk ≥10 to <15%), and high risk groups (risk ≥15).

### Intervention and measurements

Throughout the first three months of the SPRINT trial, the participants were visited every month; after that, visits occurred at 3-month intervals. Every month, drugs for the intensive treatment arm were prescribed to obtain an SBP of <120 mmHg. Differently, medications for the standard treatment arm were prescribed to obtain a systolic blood pressure of 135–139 mmHg. The dosage was reduced if the SBP was lower than 130 mmHg in one visit or below 135 mmHg in two visits. Dose adjustments were done with regards to the mean of three BP measurements in one visit. Measurements were taken with an automated system (model 907, Omron Healthcare).

Baseline demographic characteristics and the clinical and laboratory information of the subjects were gathered at first and then every three months. Structured interviews for detecting CVD outcomes were conducted in groups every three months. The participants' electrocardiographic findings and medical data were recorded. Serious adverse events (SAE) were explained as fatal or vital events, which lead to a considerable or persistent disorder, requiring a prolonged hospitalization or the researcher's judgment to establish whether or not the condition was a considerable clinical threat to the patient.

### Outcomes

Primary composite outcomes included myocardial infarction, stroke, acute decompensated heart failure, acute coronary syndrome rather than myocardial infarction, or death from cardiovascular causes. Secondary outcomes consisted of each component of the primary composite outcome separately, death from any cause, and the combination of the primary outcome or death from any reason.

### Statistical analyses

In this study, the calculated 10-year Framingham cardiovascular risk levels and the risk of developing the SPRINT primary composite cardiovascular endpoint were examined using Cox regression models. Natural cubic spline functions were applied to determine the relationship between the log hazards and BP values. All tests were two-sided and the level of significance at *p*<0.05 was considered. All analyses were performed using the Statistical Package for Social Sciences, version 17.0 (SPSS Inc., Chicago, IL, USA).

## Results

### Study population

**Fig 1:**
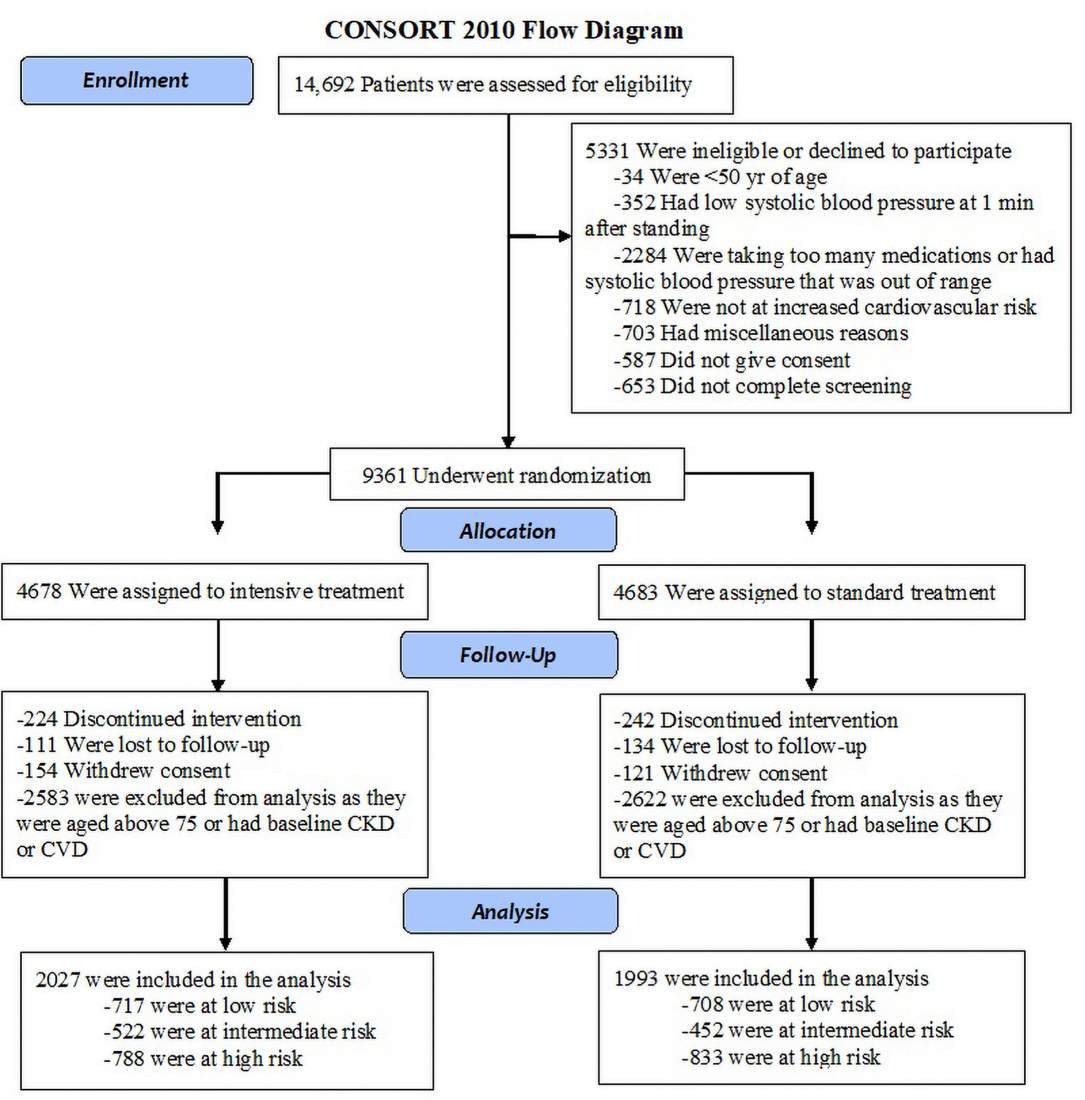
CONSORT flow diagram of the study.

In this study, 4292 participants from the SPRINT trial who met the eligibility criteria were enrolled. The flowchart of the study is presented in Fig 1. The subjects were divided into three groups according to the cardiovascular risk score. Patients were categorized into low risk (risk < 10%), intermediate risk (risk ≥10 to <15%), and high risk groups (risk ≥15) based on their 10-year Framingham cardiovascular risk scores. Characteristic features and laboratory data are provided in Table 1.

**Table 1:** Baseline characteristics of participants based on 10-year Framingham cardiovascular risk scores.

Characteristics	10-year Framingham cardiovascular risk score		
	Low risk	Intermediate risk	High risk
	risk < 10%	risk ≥10 to <15%	risk ≥15
	n = 1516	n = 1065	n = 1711
Female, n(%)	869(57.3)	327(30.7)	240(14)
Age, mean years ± SD	61.06±6.21	62.7±6.09	63.3±6.07
Race, n(%)			
Non-Hispanic black	708(46.7)	352(33.1)	356(31.3)
Hispanic	214(14.1)	161(15.1)	196(11.5)
Non-Hispanic white	568(37.5)	534(50.1)	938(54.8)
Other	26(1.7)	18(1.7)	41(2.4)
Black race, n(%)	764(50.4)	383(36)	560(32.7)
Baseline blood pressure–mmHg			
Systolic	133.5±13.26	139.4±13.72	145.34±15.7
Diastolic	79.32±10.1	81.85±10.07	84.58±11.14
Distribution of systolic blood pressure, n(%)			
< 140 mmHg	1044(68.9)	587(55.1)	658(38.5)
140–159 mmHg	426(28.1)	382(35.9)	770(45)
≥ 160 mmHg	46(3)	96(9)	283(16.5)
Serum Creatinine, mg/dl	0.9±0.18	0.94±0.18	0.96±0.17
Estimated GFR ml/min/1.73 m^2^ in those with GFR ≥60	83.07±16.57	82.27±15.42	83.18±15.86
Ratio of urinary albumin (mg) to Creatinine (g)	24.86±124.24	21.04±56.79	27.65±126.88
Fasting total Cholesterol, mg/dl	192.2±39.68	194.1±38.5	205.5±40.82
Fasting HDL cholesterol, mg/dl	56.1±15.66	51.97±12.92	48.85±12.31
Fasting total triglycerides, mg/dl	111.7±57.69	126.32±70.19	151.36±143.29
Fasting plasma glucose, mg/dl	98.68±15.32	99.56±12.58	99.65±13.85
Statin use, n (%)	541(35.9)	375(35.4)	444(26.1)
Aspirin use, n (%)	620(41)	464(43.6)	654(38.3)
Smoking status, n(%)			
Never smoked	813(53.6)	481(45.2)	593(34.7)
Former smoker	562(37.1)	440(41.3)	610(35.7)
Current smoker	141(9.3)	144(13.5)	508(29.7)
BMI	31.85±6.43	30.55±5.54	29.85±5.37
Antihypertensive agents, no./patients	2.31±0.9	1.74±0.86	1.01±0.82
Not using antihypertensive agents	9(0.6)	48(4.5)	506(29.6)

### Cardiovascular events

Analysis of the relationship between 10-year Framingham cardiovascular risk levels and the effect of intensive blood pressure reduction with risk of primary outcome is shown in Table 2. In patients with a high risk (HR, 0.45; 95% CI, 0.28 to 0.75; *p* = 0.002) or an intermediate risk (HR, 0.41; 95% CI, 0.2 to 0.84; *p* = 0.015), the primary outcome significantly occurred more in the standard treatment group, while in participants with low risk (HR, 1.33; 95% CI, 0.67 to 2.65; *p* = 0.422), the primary outcome happened non-significantly more in the intensive treatment group.

**Table 2:** Primary outcome based on stratified cardiovascular risk scores.

10-year Framingham cardiovascular risk score	Intensive treatment group	Standard treatment group	HR (CI)	P value
Low, n(%)	19(13.5)	14(9.9)	1.33(0.67–2.65)	0.422
Intermediate, n(%)	11(7.8)	24(17)	0.41(0.2–0.84)	0.015
High, n(%)	22(15.6)	51(36.2)	0.45(0.28–0.75)	0.002

**Fig 2:**
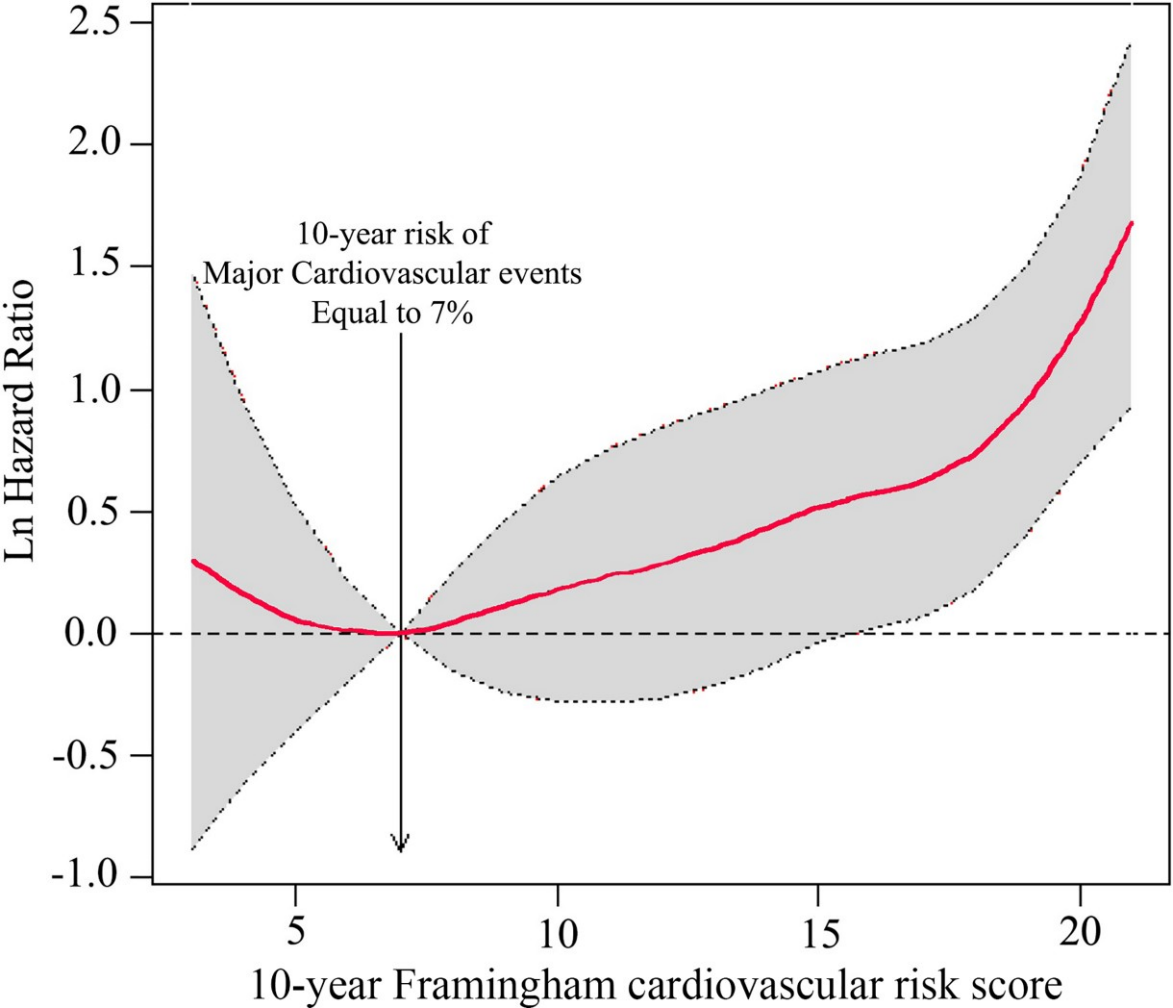
Natural cubic spline curve demonstrating a J-shaped relationship between efficacy of intensive blood pressure reduction and 10-year Framingham cardiovascular risk.

Natural cubic spline functions were applied to determine the relationship between the log hazards and BP values. Analysis results revealed a clear J-shaped relationship between 10-year Framingham cardiovascular risk levels and the effect of intensive blood pressure reduction with risk of fatal and non-fatal CVD events at a threshold of approximately <7% (Fig 2).

**Fig 3:**
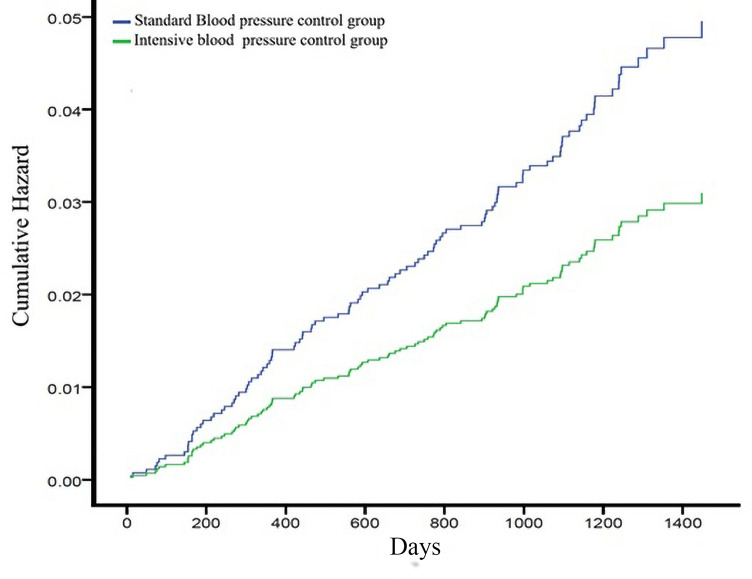
Cumulative hazards for the primary outcome in intensive and standard treatment groups in participants with a 10-year Framingham cardiovascular risk score >7%.

Cumulative hazards for the primary outcome in intensive and standard treatment groups in participants with a 10-year Framingham cardiovascular risk score >7% are shown in Fig 3.

### Clinical outcomes

Primary and secondary outcomes in patients with a 10-year Framingham cardiovascular risk score above 7 are shown in Table 3. Occurrence of the primary outcome was significantly lower (HR = 0.51; CI, 0.35 to 0.74; *p*<0.001) in the intensive therapy group. Furthermore, patients with intensive therapy showed a significant benefit in stroke (HR = 0.38; CI, 0.18 to 0.81; *p* = 0.013), death from cardiovascular causes (HR = 0.25; CI, 0.07 to 0.89; *p* = 0.033), and primary outcome and death (HR = 0.57; CI, 0.42 to 0.78; *p* = 0.001).

**Table 3:** Primary and secondary outcomes in patients with a 10-year Framingham cardiovascular risk score >7%.

	Intensive treatment group, n(%)	Standard treatment group, n(%)	HR(CI)	P value
	n = 1778	n = 1790		
Primary Outcome	42(2.4)	82(4.6)	0.51(0.35–0.74)	<0.001
Secondary Outcomes				
Myocardial Infarction	21(1.2)	35(2)	0.6(0.35–1.04)	0.068
Acute Coronary Syndrome	5(0.3)	7(0.4)	0.7(0.23–2.27)	0.575
Stroke	9(0.5)	24(1.3)	0.38(0.18–0.81)	0.013
Heart Failure	8(0.4)	15(0.8)	0.54(0.23–1.27)	0.156
Death from cardiovascular causes	3(0.2)	12(0.7)	0.25(0.07–0.89)	0.033
Death from any cause	28(1.6)	42(2.3)	0.67(0.42–1.09)	0.106
Primary outcome or death	60(3.4)	105(5.9)	0.57(0.42–0.78)	0.001

Intensive blood pressure reduction in patients with a risk score >7 had no effect on sex, race, baseline blood pressure level, or taking aspirin or statins, as demonstrated in Table 4.

**Table 4:** Primary outcome based on subgroups in those with a 10-year Framingham cardiovascular risk score >7%.

	Intensive treatment group	Standard treatment group	HR(CI)	P value of interaction
Sex				
Female	12/939(1.3)	17/939(1.8)	0.69(0.33–1.44)	0.374
Male	30/2629(1.1)	65/2629(2.5)	0.46(0.3–0.72)	
SBP				
<140 mmHg	20/1736(1.1)	40/1736(2.3)	0.49(0.28–0.83)	0.472
140–159 mmHg	15/1424(1)	34/1424(2.4)	0.45(0.25–0.83)	
≥160 mmHg	7/408(1.7)	8/408(2)	0.87(0.32–2.4)	
Race				
Black	14/1315(1.1)	30/1315(2.3)	0.49(0.26–0.92)	0.842
Non-black	28/2253(1.2)	52/2253(2.3)	0.52(0.33–0.83)	
Aspirin				
Yes	16/1442(1.1)	35/1442(2.4)	0.45(0.25–0.81)	0.571
No	26/2120(1.2)	47/2120(2.2)	0.56(0.35–0.9)	
Statin				
Yes	16/1107(1.4)	33/1107(3)	0.5(0.27–0.91)	0.914
No	26/2441(1.1)	49/2441(2)	0.52(0.32–0.84)	

### Serious adverse events

Overall serious adverse events were observed more in the group with intensive therapy (528 cases) compared to standard therapy (485 cases) (HR = 1.21; 95% CI, 0.99–1.27; *p* = 0.07). Furthermore, acute kidney injury or acute kidney failure occurred significantly more in the group with intensive therapy (HR, 2.76; 95% CI, 1.58 to 4.81; *p*<0.001). The adverse events are reported in detail in Table 5.

**Table 5:** Serious adverse events, condition of interest and monitored clinical events in participants with a 10-year Framingham cardiovascular risk score> 7.

	Intensive treatment group	Standard treatment group	HR	P value
Serious Adverse Events	528(29.7)	485(27.1)	1.21(0.99–1.27)	0.07
Condition of Interest				
Serious Adverse Events only				
Hypotension	26(1.5)	14(0.8)	1.88(0.98–3.6)	0.057
Syncope	29(1.6)	21(1.2)	1.4(0.8–2.46)	0.237
Bradycardia	16(0.9)	9(0.5)	1.81(0.8–4.1)	0.154
Electrolyte abnormality	26(1.5)	33(1.8)	0.79(0.48–1.33)	0.383
Injurious fall	15(0.8)	16(0.9)	0.95(0.47–1.92)	0.888
Acute Kidney Injury or Acute Kidney Failure	46(2.6)	17(0.9)	2.76(1.58–4.81)	<0.001
Emergency department visit or Serious adverse events				
Hypotension	46(2.6)	20(1.1)	2.34(1.39–3.96)	0.001
Syncope	50(2.8)	31(1.7)	1.64(1.05–2.57)	0.03
Bradycardia	19(1.1)	9(0.5)	2.15(0.97–4.75)	0.058
Electrolyte abnormality	40(2.2)	39(2.2)	1.04(0.67–1.61)	0.871
Injurious fall	72(4)	67(3.7)	1.09(0.78–1.52)	0.612
Acute Kidney Injury or Acute Kidney Failure	51(2.9)	18(1)	2.9(1.69–4.96)	<0.001
Monitored Clinical events				
Adverse lab measures				
Serum Sodium < 130	63(3.5)	28(1.6)	2.31(1.48–3.61)	<0.001
Serum Sodium >150	2(0.1)	0		0.47
Serum Potassium < 3	46(2.6)	33(1.8)	1.43(0.92–2.24)	0.116
Serum Potassium > 5.5	40(2.2)	41(2.3)	1(0.64–1.54)	0.992
Orthostatic hypotension				
Alone	248(13.9)	281(15.7)	0.9(0.76–1.07)	0.232
With dizziness	16(0.9)	25(1.4)	0.65(0.35–1.23)	0.185

## Discussion

This study is the first to analyze the presence of a J-shaped curve relationship between baseline cardiovascular risk and the effect of intensive blood pressure reduction. The 2017 ACC/AHA hypertension guidelines recommend initiation of antihypertensive medication at lower thresholds (130/80 mmHg) and lower blood pressure target goals [6]. Questions regarding the target goal of hypertension treatment [12] in the 2017 ACC/AHA guidelines and criticism that a risk-based strategy is not based on randomized, controlled trials [7] prompted the performance of a secondary analysis of the SPRINT trial to find the relationship between cardiovascular risk and the effect of intensive blood pressure reduction. The results revealed a clear J-shaped relationship between 10-year Framingham cardiovascular risk levels and the effect of intensive blood pressure reduction with risk of fatal and non-fatal CVD events at a threshold of approximately <7%. Despite not being based on ASCVD risk scores; it may still be shown that a lower threshold than the current ACC/AHA recommendation level should be chosen for starting intensive blood pressure reduction.

Using a risk-based strategy for controlling blood pressure has several benefits. First of all, studies have shown that cardiovascular events happen more among hypertensive patients with a high cardiovascular risk than those with a low cardiovascular risk [9–11]. Karmali et al. in a pooled analysis showed that most cardiovascular events occurred in participants with SBP<140 and DBP<90, while 65% of events happened among those with a 10-year cardiovascular disease risk more than 7.5% [13]. Furthermore, treatment of blood pressure based on a cardiovascular disease risk is associated with less visit-to-visit variability and measurement error than that based on blood pressure alone. Ye et al. found that 10-year predicted cardiovascular disease risk alterations are fewer than changes in blood pressure [14]. Moreover, a risk-based strategy helps physicians [15] have better judgment in treating hypertensive patients.

There is some controversy in comparing the current results with other studies. In a post hoc analysis of the SPRINT, Ling Zhang et al. concluded that regardless of 10-year Framingham cardiovascular risk levels, intensive blood pressure reduction was helpful [16]. Their study design and population can somewhat explain the difference in results. For example, they enrolled participants with cardiovascular disease and chronic kidney disease. Additionally, in their meta-analysis, Thomopoulos et al. concluded that cardiovascular risk should not be considered for lowering systolic blood pressure to less than 130 mmHg [17]. This controversy could be due to different target goals and risk stratification. Actually, the meta analysis included a trivial number of studies with target goals of SBP less than 120 mm Hg; the current study considered a higher and different cut-off limit for cardiovascular risk than theirs. In another meta-analysis, Böhm et al. assessed high-risk participants older than 55 years of age with a prior history of cardiovascular disease from the ONTARGET [18] and TRANSCEND [19] trials. The results demonstrated lower mortality risk, and cardiovascular events were associated with an SBP target around 130 mmHg, while in an SBP less than 120 mmHg, cardiovascular events other than myocardial infarction and stroke were increased [20]. The study design and population could clarify the differences between the results of the current study and theirs. Diabetic participants were included in these trials, but not in the SPRINT. In contrast to the ONTARGET and TRANSCEND trials, blood pressure measuring in the SPRINT was unattended, which could cause values 5 to 10 mmHg lower than those routinely obtained [21]. The Heart Outcomes Prevention Evaluation (HOPE)–3 trial [22] consisted of 12,705 participants at intermediate cardiac risk and demonstrated that antihypertensive treatment was not beneficial for decreasing cardiovascular events. Receiving medications for the control group, defining primary outcome, risk stratification, study population, and blood pressure target in the SPRINT were different than those in HOPE-3, which made comparisons difficult.

The advantages of intensive treatment are associated with some adverse effects. Serious adverse events (SAEs) due to hypotension, syncope, and acute kidney injury or acute renal failure occurred more in the intensive treatment arm. No significant difference in injurious fall was found, although hypotension and syncope happened more frequently in the intensive-treatment group.

Several limitations in this study should be noted. Due to excluding participants with low or intermediate risks in the original protocol of the SPRINT, stratified randomization of patients with these risks was not done properly, and the sample size of these groups were quite small. The SPRINT participants were almost all previously diagnosed as having hypertension and on anti-hypertensive medications which affected risk-based classification. In addition, in the low risk group, the factors influencing CVD risk other than hypertension were increased. On the other hand, the small number of patients/events, particularly in the low risk group, decreased the statistical power of this analysis. These can justify to some extent the greater HR for the primary outcome in the low risk group compared to the intermediate and high risk groups. Furthermore Berkelmans et al. revealed that the possibility of changes in blood pressure could not explain all the positive findings in the SPRINT trial. Other explanations should be considered, such as dissimilarity in therapy strategies. For instance, higher rates of diuretic use in the intensive treatment group could also account for the positive effects in the SPRINT trial [23], as low-dose diuretics are beneficial for preventing cardiovascular diseases in heart failure patients [24–27]. It seems further investigations are required to answer the questions regarding a risk-based strategy for treating hypertension.

In conclusion, as the threshold for starting statins is considered as 7.5%, choosing the same level for selecting patients who have gains from intensive blood pressure reduction may simplify preventive cardiology rules. However, this conclusion should be considered with some precaution, as it is based on a post hoc analysis, and has used Framingham risk score instead of ASCVD risk.

## References

[1] Bromfield S, Muntner P. High blood pressure: the leading global burden of disease risk factor and the need for worldwide prevention programs. Curr Hypertens Rep. 2013;15(3):134–6. Epub 2013/03/29. 10.1007/s11906-013-0340-9 23536128 PMC3699411

[2] James PA, Oparil S, Carter BL, Cushman WC, Dennison-Himmelfarb C, Handler J, et al 2014 evidence-based guideline for the management of high blood pressure in adults: report from the panel members appointed to the Eighth Joint National Committee (JNC 8). Jama. 2014;311(5):507–20. Epub 2013/12/20. 10.1001/jama.2013.284427 . 24352797

[3] Avanzini F, Marzona I, Baviera M, Barlera S, Milani V, Caimi V, et al Improving cardiovascular prevention in general practice: Results of a comprehensive personalized strategy in subjects at high risk. Eur J Prev Cardiol. 2016;23(9):947–55. Epub 2015/11/04. 10.1177/2047487315613664 . 26525065

[4] Joint British Societies' consensus recommendations for the prevention of cardiovascular disease (JBS3). Heart. 2014;100 Suppl 2:ii1–ii67. Epub 2014/03/29. 10.1136/heartjnl-2014-305693 . 24667225

[5] Stone NJ, Robinson JG, Lichtenstein AH, Bairey Merz CN, Blum CB, Eckel RH, et al 2013 ACC/AHA guideline on the treatment of blood cholesterol to reduce atherosclerotic cardiovascular risk in adults: a report of the American College of Cardiology/American Heart Association Task Force on Practice Guidelines. Circulation. 2014;129(25 Suppl 2):S1–45. Epub 2013/11/14. 10.1161/01.cir.0000437738.63853.7a . 24222016

[6] Whelton PK, Carey RM, Aronow WS, Casey DE Jr., Collins KJ, Dennison Himmelfarb C, et al 2017 ACC/AHA/AAPA/ABC/ACPM/AGS/APhA/ASH/ASPC/NMA/PCNA Guideline for the Prevention, Detection, Evaluation, and Management of High Blood Pressure in Adults: A Report of the American College of Cardiology/American Heart Association Task Force on Clinical Practice Guidelines. J Am Coll Cardiol. 2018;71(19):e127–e248. Epub 2017/11/18. 10.1016/j.jacc.2017.11.006 . 29146535

[7] Bakris G, Sorrentino M. Redefining Hypertension—Assessing the New Blood-Pressure Guidelines. N Engl J Med. 2018;378(6):497–9. Epub 2018/01/18. 10.1056/NEJMp1716193 . 29341841

[8] Wright JT Jr., Williamson JD, Whelton PK, Snyder JK, Sink KM, Rocco MV, et al A Randomized Trial of Intensive versus Standard Blood-Pressure Control. N Engl J Med. 2015;373(22):2103–16. Epub 2015/11/10. 10.1056/NEJMoa1511939 26551272 PMC4689591

[9] Attar A, Sayadi M, Jannati M. Effect of intensive blood pressure lowering on cardiovascular outcomes based on cardiovascular risk: A secondary analysis of the SPRINT trial. Eur J Prev Cardiol. 2019;26(3):238–45. Epub 2018/09/27. 10.1177/2047487318800741 . 30256671

[10] Karmali KN, Lloyd-Jones DM, van der Leeuw J, Goff DC Jr., Yusuf S, Zanchetti A, et al Blood pressure-lowering treatment strategies based on cardiovascular risk versus blood pressure: A meta-analysis of individual participant data. PLoS Med. 2018;15(3):e1002538 Epub 2018/03/21. 10.1371/journal.pmed.1002538 29558462 PMC5860698

[11] Muntner P, Whelton PK. Using Predicted Cardiovascular Disease Risk in Conjunction With Blood Pressure to Guide Antihypertensive Medication Treatment. J Am Coll Cardiol. 2017;69(19):2446–56. Epub 2017/05/13. 10.1016/j.jacc.2017.02.066 28494981 PMC5873607

[12] Kaul S. How Strong Is the Evidence to Support Blood Pressure Treatment Goal of 130/80 mm Hg? Circulation. 2018;138(23):2594–6. Epub 2018/12/21. 10.1161/CIRCULATIONAHA.118.037669 . 30571265

[13] Karmali KN, Ning H, Goff DC, Lloyd-Jones DM. Identifying Individuals at Risk for Cardiovascular Events Across the Spectrum of Blood Pressure Levels. J Am Heart Assoc. 2015;4(9):e002126 Epub 2015/09/24. 10.1161/JAHA.115.002126 26391134 PMC4599500

[14] Ye S, Wang YC, Shimbo D, Newman JD, Levitan EB, Muntner P. Effect of change in systolic blood pressure between clinic visits on estimated 10-year cardiovascular disease risk. J Am Soc Hypertens. 2014;8(3):159–65. Epub 2014/01/28. 10.1016/j.jash.2013.12.006 24462238 PMC3959282

[15] Pignone M, Phillips CJ, Elasy TA, Fernandez A. Physicians' ability to predict the risk of coronary heart disease. BMC Health Serv Res. 2003;3(1):13 Epub 2003/07/15. 10.1186/1472-6963-3-13 12857356 PMC183837

[16] Zhang L, Sun X, Liao L, Zhang S, Zhou H, Zhong X, et al Effectiveness of blood pressure-lowering treatment by the levels of baseline Framingham risk score: A post hoc analysis of the Systolic Blood Pressure Intervention Trial (SPRINT). J Clin Hypertens (Greenwich). 2019;21(12):1813–20. Epub 2019/11/02. 10.1111/jch.13720 . 31670874 PMC8030550

[17] Thomopoulos C, Parati G, Zanchetti A. Effects of blood-pressure-lowering treatment on outcome incidence in hypertension. 11. Effects of total cardiovascular risk and achieved blood pressure: overview and meta-analyses of randomized trials. J Hypertens. 2017;35(11):2138–49. Epub 2017/09/01. 10.1097/HJH.0000000000001548 . 28858983

[18] Yusuf S, Teo KK, Pogue J, Dyal L, Copland I, Schumacher H, et al Telmisartan, ramipril, or both in patients at high risk for vascular events. N Engl J Med. 2008;358(15):1547–59. Epub 2008/04/02. 10.1056/NEJMoa0801317 . 18378520

[19] Yusuf S, Teo K, Anderson C, Pogue J, Dyal L, Copland I, et al Effects of the angiotensin-receptor blocker telmisartan on cardiovascular events in high-risk patients intolerant to angiotensin-converting enzyme inhibitors: a randomised controlled trial. Lancet. 2008;372(9644):1174–83. Epub 2008/09/02. 10.1016/S0140-6736(08)61242-8 . 18757085

[20] Böhm M, Schumacher H, Teo KK, Lonn EM, Mahfoud F, Mann JFE, et al Achieved blood pressure and cardiovascular outcomes in high-risk patients: results from ONTARGET and TRANSCEND trials. Lancet. 2017;389(10085):2226–37. Epub 2017/04/10. 10.1016/S0140-6736(17)30754-7 . 28390695

[21] Wohlfahrt P, Cífková R, Movsisyan N, Kunzová Š, Lešovský J, Homolka M, et al Threshold for diagnosing hypertension by automated office blood pressure using random sample population data. J Hypertens. 2016;34(11):2180–6. Epub 2016/08/12. 10.1097/HJH.0000000000001076 . 27512968

[22] Lonn EM, Bosch J, López-Jaramillo P, Zhu J, Liu L, Pais P, et al Blood-Pressure Lowering in Intermediate-Risk Persons without Cardiovascular Disease. N Engl J Med. 2016;374(21):2009–20. Epub 2016/04/05. 10.1056/NEJMoa1600175 . 27041480

[23] Berkelmans GF, Visseren FL, Jaspers NE, Spiering W, van der Graaf Y, Dorresteijn JA. SPRINT trial: It's not just the blood pressure! Eur J Prev Cardiol. 2017;24(14):1482–4. Epub 2017/07/28. 10.1177/2047487317723213 . 28749177

[24] Psaty BM, Lumley T, Furberg CD, Schellenbaum G, Pahor M, Alderman MH, et al Health outcomes associated with various antihypertensive therapies used as first-line agents: a network meta-analysis. Jama. 2003;289(19):2534–44. Epub 2003/05/22. 10.1001/jama.289.19.2534 . 12759325

[25] Attar A, Sadeghi AA, Amirmoezi F, Aghasadeghi K. Low Dose Spironolactone Monotherapy in the Management of Stage I Essential Hypertension: A Pilot Randomized, Double-Blind, Placebo-Controlled Trial. Acta Cardiol Sin. 2018;34(1):59–65. Epub 2018/01/30. 10.6515/ACS.201801_34(1).20170903B 29375225 PMC5777944

[26] Attar A, Sayadi M. Effect of Chronic Kidney Disease on Cardiovascular Events: An Epidemiological Aspect from SPRINT Trial. Iran J Kidney Dis. 2019;13(5):328–36. Epub 2019/11/11. . 31705750

[27] Sayadi M, Zare N, Attar A, Ayatollahi SMT. Improved Landmark Dynamic Prediction Model to Assess Cardiovascular Disease Risk in On-Treatment Blood Pressure Patients: A Simulation Study and Post Hoc Analysis on SPRINT Data. Biomed Res Int. 2020;2020:2905167 Epub 2020/05/10. 10.1155/2020/2905167 32382541 PMC7195630

